# Metformin use improves survival of diabetic liver cancer patients: systematic review and meta-analysis

**DOI:** 10.18632/oncotarget.11033

**Published:** 2016-08-02

**Authors:** Shu-Juan Ma, Yi-Xiang Zheng, Peng-Cheng Zhou, Yan-Ni Xiao, Hong-Zhuan Tan

**Affiliations:** ^1^ Department of Epidemiology and Health Statistics, Xiangya School of Public Health, Central South University, Changsha, China; ^2^ Department of Infectious Disease, Viral Hepatitis Key Laboratory of Hunan Province, Xiangya Hospital, Central South University, Changsha, China

**Keywords:** metformin, liver cancer, survival, diabetes mellitus, meta-analysis

## Abstract

Metformin has garnered considerable interest as a chemo-preventive and chemo-therapeutic agent given the increased risk of liver cancer among diabetic patients. This work was performed to illustrate the association between metformin use and survival of diabetic liver cancer patients. We conducted a comprehensive literature search of PubMed, Web of Science, Embase, BIOSIS Previews, Cochrane Library from inception to 12 May 2016. Meta-analyses were performed using Stata (version 12.0), with hazard ratios (HRs) and corresponding 95% confidence intervals (CIs) as effect measures. Eleven cohort studies involving 3452 liver cancer patients fulfilled the inclusion criteria. Meta-analyses showed that metformin use was associated with better survival (HR = 0.59; 95% CI, 0.42-0.83; *p* = 0.002) of liver cancer patients, and the beneficial effect persisted (HR = 0.64; 95% CI, 0.42-0.97; *p* = 0.035) when the population was restricted to diabetic liver cancer patients. After adjusting for age, etiology, index of tumor severity and treatment of liver cancer, the association between metformin use and better survival of liver cancer patients was stable, pooled HR ranged from 0.47 to 0.57. The results indicated that metformin use improved survival of diabetic liver cancer patients. However, the results should be interpreted with caution given the possibility of residual confounding. Further prospective studies are still needed to confirm the prognostic benefit of metformin use.

## INTRODUCTION

Liver cancer is one of the leading malignancies worldwide, with an overall 5-year survival rate of less than 15% [1]. Diabetes mellitus (DM), an increasingly common chronic disease, is frequently encountered with liver cancer in clinical practice, probably because of the shared risk factors [2]. Epidemiological and clinical evidence has linked DM to the poor prognosis of many cancers through multifactorial mechanisms [3-5]. However, this effect may be mitigated by anti-diabetic medications (ADMs) [6, 7]. Metformin, one of the most commonly prescribed ADMs, has received great attention for its anti-tumor activity. Accumulating studies have researched the role of metformin in both cancer prevention and treatment. Survival benefits of metformin have been demonstrated in a wide range of malignancies including breast cancer, prostate cancer, pancreatic cancer, colorectal cancer and lung cancer, through corresponding meta-analyses [8-12]. However, only one previous meta-analysis involving two related studies summarized evidence on survival effect of metformin in liver cancer patients [13]. It is still uncertain whether use of metformin could also generate better clinical outcomes for patients with liver cancer.

Preclinical studies have demonstrated indirect and direct beneficial effects of metformin on cultured human hepatocellular carcinoma (HCC) cell lines, in xenograft tumors model *in vivo*, and on animal livers [14-17]. Simultaneously, a growing number of observational studies have compared metformin with non-metformin treatment on prognostic outcomes of liver cancer patients, showing somewhat inconsistent results [18-20]. Given that understanding the efficacy of metformin in liver cancer treatment may lead to better clinical management, we embarked a systematic review and meta-analysis to illustrate the association of metformin use with survival of liver cancer patients.

## RESULTS

### Description of included studies

**Figure 1:**
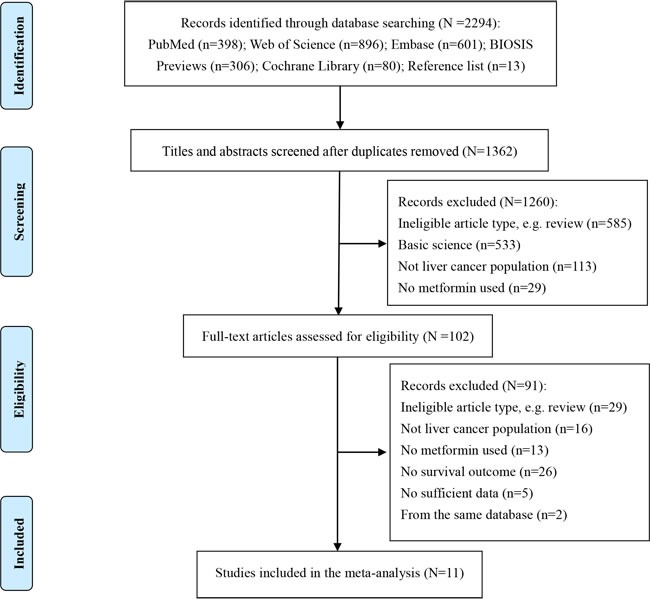
Flow diagram of study selection

The flow diagram for study selection is shown in Figure 1. Of the 2294 titles identified, 1362 abstracts and 102 resulting full-text studies were reviewed to determine their eligibility. To avoid overlapping patient populations, two overlapped studies [21, 22] were excluded. Finally, 11 cohort studies [18-20, 23-30] and 3452 liver cancer patients were included in our overall analysis of the effect of metformin on survival of liver cancer patients.

The characteristics of included cohort studies are listed in Table 1. Majority of studies were retrospective design, clinic-based setting and conducted in western countries. Quality of 7 studies was high based on the Newcastle-Ottawa Scale (NOS) [18-20, 24, 27, 28, 30]. Liver cancer populations of 8 studies [18-20, 23, 25, 26, 29, 30] were limited to DM patients, and others [24, 27, 28] were without this restriction. Meanwhile, 3 studies [18-20] also compared survival of diabetic metformin users with non-diabetic non-metformin users. Seven studies [18, 19, 24, 27-30] reported estimations defining the metformin exposure as taking metformin after the diagnosis of liver cancer, including those [18, 19, 29] taking metformin on the date of diagnosis and continued during the follow-up period. Meanwhile 4 studies [20, 23, 24, 30] reported estimations defining as taking metformin before the cancer diagnosis. In addition to age [18-20, 23-25, 27-30], most studies adjusted for etiology [18-20, 23, 25, 28, 30], index of tumor severity [18, 19, 23, 25, 27-29] and treatment of liver cancer [18, 20, 23, 25, 28, 30].

**Table:** Characteristics of studies included in the meta-analysis

Study (year)	Design	Location	NOS score	Data source	Time period	Study population	Definition of metformin exposure	Total subject	Mean follow-up	Adjustment variables
Chen 2011 [18]	Retrospective cohort	China	7	Tungs' Taichung MetroHarbor Hospital	2003.07-2010.09	Early stage (BCLC stage 0 or A) HCC patients with DM after RFA	On the date of HCC occurrence and periods during follow-up	53	32.2 months	Age, sex, BMI, HbA_1c_, anti-HCV antibody, and tumor size (cut-off at 2.5 cm)
Akmal 2012 [23]	Cohort	USA	3	NR	2001-2010	HCC patients with DM	More than 1 year prior to HCC diagnosis	130	NR	Age, HCV infection, alcohol consumption, treatment of HCC, cirrhosis history, Liver Italian Program staging score
Currie 2012 [24]	Retrospective cohort	U.K	9	Primary care practices	1990-2009.12	Liver cancer patients	In the 90 days before liver cancer diagnosis	1460	1.6 years	Age, sex, smoking history, Townsend index of deprivation , Charlson comorbidity index , number of primary care contacts, year of diagnosis
Hassabo 2012 [25]	Cohort	USA	3	NR	2000-2012	HCV-induced HCC patients with DM	NR	56	NR	Age, sex, race, cirrhosis, AFP, prior treatment, staging
Graef 2013 [26]	Prospective cohort	U.K	3	NR	2007-2012	HCC patients with DM	NR	282	NR	NR
Ampuero 2014 [27]	Cohort	Spain	5	Surveillance program	2005-2013	Cirrhotic patients with HCC	After HCC diagnosis	125	1.8 years	Age, diffuse HCC, multinodular HCC, statins use, nodule > 5 cm, vascular invasion, metastasis
Bhat 2014 [19]	Retrospective cohort	USA	6	Mayo Clinic	2005.01-2011.06	HCC patients with DM	At time of HCC diagnosis and continued beyond 90 days following diagnosis	263	NR	Age, sex, caucasian, etiologies of liver disease, BCLC stage
Casadei 2015 [20]	Retrospective cohort	Italy	6	Medical records databases of IRST IRCCS	2008.03-2014.08	HCC patients with DM consecutively treated with sorafenib twice daily	On metformin for at least 5 years when HCC was diagnosis	42	NR	Age, sex, smoking habits and etiology
Jang 2015 [28]	Retrospective cohort	Korea	7	Four institutions	2003.03-2012.12	HCC patients who were treated with SBRT or HypoRT	Received metformin for at least 1 year during radiotherapy	76	15 months	Age, sex, diabetic status, ECOG PS, etiology, number of TACE, PVTT, BED, tumor size, Child-Pugh class, AFP level, multiple tumor lesions
Yang 2015 [29]	Retrospective cohort	USA	4	Mayo Clinic	2001.01-2012.12	Newly diagnosed CCA patients with DM	On the date of CCA diagnosis and continued after diagnosis	214	24.7 months	Age, sex, smoking, PSC, ECOG, CA19-9, tumor size, vascular encasement, metastasis
Seo 2016 [30]	Retrospective cohort	South Korea	9	NHIS and KCCR	2005.01-2011.12	HCC patients with DM who undergone curative hepatic resection	Received in the same class for ≥ 90 days during the follow-up period	751	NR	Age, sex, hepatitis type, antiviral medication, and Charlson comorbidity index

NOTE: Abbreviations: AFP, α-fetoprotein; BCLC, Barcelona clinic liver cancer; BED, biologically equivalent dose; BMI, body mass index; CA19-9, cancer antigen 19-9; CCA, cholangiocarcinoma; DM, diabetes mellitus; ECOG PS, Eastern Cooperative Oncology Group performance status; HbA_1c_, hemoglobin A_1c_; HBV, hepatitis B virus; HCC, hepatocellular cancer; HCV, hepatitis C virus; HypoRT, hypofractionated radiotherapy; KCCR, Korea Center Cancer Registry; NHIS, National Health Insurance Service; NOS, Newcastle-Ottawa Scale; NR, not reported; NSAIDs, nonsteroidal antiinflammatory drugs; PSC, primary sclerosing cholangitis; PVTT, portal vein tumor thrombus; RFA, radiofrequency ablation; SBRT, stereotactic body radiotherapy; TACE, transarterial chemoembolization.

### Overall analysis

**Figure 2:**
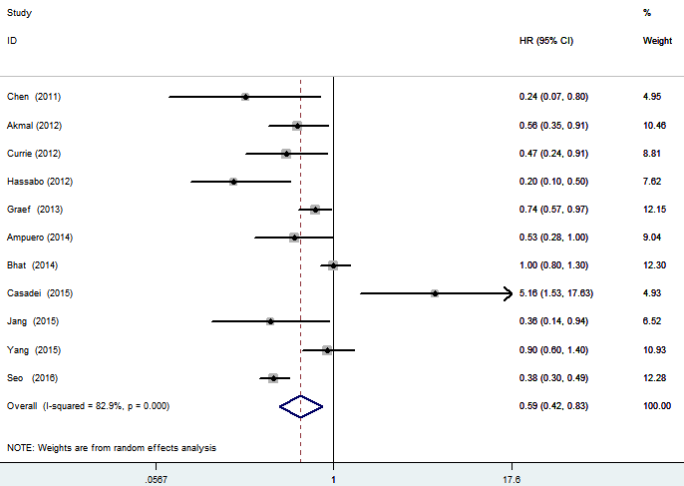
Forest plot of the association between metformin use and survival of liver cancer patients

Pooled hazard ratios (HRs) and corresponding 95% confidence intervals (CIs) are shown in Figure 2. Meta-analysis of 11 studies [18-20, 23-30] showed that metformin use was associated with a 41% significant decreased mortality in 3452 liver cancer patients (HR = 0.59; 95% CI, 0.42-0.83; *p* = 0.002), with high heterogeneity (I^2^ = 82.9%). Sensitivity analysis found that the high heterogeneity was not due to any single study, summary result was demonstrated to be robust through leave-one-out method. Meta-regression analysis found that publication year (*p* = 0.279), location (*p* = 0.168), NOS score (*p* = 0.744) and number of total subject (*p* = 0.671) failed to account for heterogeneity in any of the preplanned comparisons. No significant publication bias was found, neither from Begg's test (*p* = 0.276) nor from Egger's test (*p* = 0.676).

### Subgroup analyses

Subgroup analyses were performed to further explore potential sources of the high heterogeneity among studies and validate the result from overall analysis (Table 2). Stratified analyses by study quality found that the decreased mortality in metformin users lost significance in 7 high quality studies (HR = 0.61; 95% CI, 0.35-1.05; *p* = 0.072). Subgroup analysis of 3 Asian studies showed an exaggeration in metformin's effect (HR = 0.37; 95% CI, 0.30-0.47; *p* < 0.001). When defined the exposure as taking metformin before cancer diagnosis, the beneficial effect on survival lost significance (HR = 0.69; 95% CI, 0.37-1.29; *p* = 0.249). When the controlled non-metformin users were restricted to liver cancer patients with DM, the beneficial effect for metformin use was stable (HR = 0.64; 95% CI, 0.42-0.97; *p*= 0.035), and persisted in controlled population without this restriction (HR = 0.47; 95% CI, 0.31-0.71; *p* < 0.001). However, when compared to non-diabetic non-metformin users, diabetic metformin users showed worse survival (HR = 1.35; 95% CI, 0.99-1.82; *p* = 0.054). After adjusting for age, etiology, index of tumor severity and treatment of liver cancer, the association between metformin use and better survival of liver cancer patients was stable, pooled HRs (95% CIs) were 0.57 (0.38-0.85), 0.55 (0.31-0.96), 0.54 (0.35-0.82) and 0.47 (0.27-0.84), respectively.

**Table 2:** Summary results of subgroup analyses of association between metformin use and survival of liver cancer patients

Subgroup	No. of studies	Total subject	Summary result		*I*^2^ (%)
			HR (95% CI)	*P* value	
**Quality**					
High	7	2770	0.61 (0.35-1.05)	0.072	87.0
Low	4	682	0.59 (0.38-0.91)	0.018	74.1
**Location**					
Asian	3	880	0.37 (0.30-0.47)	< 0.001	0
Western	8	2572	0.70 (0.49-0.98)	0.041	76.9
**Definition of metformin**					
Before cancer diagnosis	4	2084	0.69 (0.37-1.29)	0.249	79.8
After cancer diagnosis^#^	7	2301	0.60 (0.39-0.93)	0.023	84.4
**Controlled population**					
DM	8	1791	0.64 (0.42-0.97)	0.035	87.5
DM + Non-DM	3	1661	0.47 (0.31-0.71)	< 0.001	0
Non-DM	3	679	1.35 (0.99-1.82)	0.054	20.4
**Adjustment**					
Age	10	3170	0.57 (0.38-0.85)	0.007	84.2
Etiology	7	1371	0.55 (0.31-0.96)	0.037	88.6
Index of tumor severity	7	917	0.54 (0.35-0.82)	0.004	75.7
Treatment of liver cancer	6	1108	0.47 (0.27-0.84)	0.011	77.6

Note: Abbreviations: CI, confidence interval; DM, diabetes mellitus; Non-DM, non-diabetes mellitus; HR, hazard ratio.

# included those taking metformin on the date of diagnosis and continued during the follow-up period.

Significant heterogeneity was present in almost all the subgroups, with *I*^2^ (> 50%) ranging from 74.1% to 88.6% (Table 2). No heterogeneity was found in the subgroup analysis of 3 Asian studies (*I*^2^ = 0). Moreover, when the controlled population did not restrict to DM patients, the heterogeneity disappeared (*I*^2^ = 0). And when the controlled population restricted to non-DM patients, the heterogeneity was limited (*I*^2^ = 20.4%).

## DISCUSSION

In this meta-analysis of 11 cohort studies and 3452 liver cancer patients, we found that, relative to non-use, use of metformin significantly reduced mortality (HR = 0.59; 95% CI, 0.42-0.83; *p* = 0.002). This significant effect was validated in most subgroup analyses. However, stratified analyses by study quality got a conflicting result that the decreased mortality in metformin users lost significance in 7 high quality studies (HR = 0.61; 95% CI, 0.35-1.05; *p* = 0.072). Referring back to the original studies, one study by Casadei et al [20] might be the outlier, which reported a significant unfavorable effect of metformin in DM patients with advanced HCC receiving sorafenib (HR = 5.16; 95% CI, 1.53-17.63; *p* = 0.008). It was greatly different from the beneficial effects reported in most other studies. After excluding this suspicious outlier, pooled result of remaining 6 high quality studies further validated the significant beneficial effect of metformin (HR = 0.49; 95% CI, 0.29-0.81; *p* = 0.006). More accurately from a clinical point of view, this study suggested that metformin did not enhance the activity of sorafenib during the development of HCC. However, a synergistic effect against HCC was found between metformin and radiotherapy [18, 28]. Synergistic benefits were also found between metformin and chemotherapy/radiotherapy against certain cancer types [31-35]. The interactions may be related to their molecular mechanisms.

Although the anti-tumor action of metformin has been reported by accumulating preclinical *in vitro* and *in vivo* studies [16], the potential molecular mechanism has still not been fully elucidated [36]. The mechanisms are mainly divided into indirect effects by reducing circulating glucose or insulin levels, and direct effects on tumor cells through adenosine monophosphate-activated protein kinase (AMPK)-dependent and AMPK-independent mechanisms [37, 38]. Sorafenib has been reported to act through the same AMPK activation pathway as metformin [39]. Thus a possible explanation for sorafenib-resistance is that, tumors are more likely to have intrinsic mechanisms of resistance to metformin during chronic treatment with it, which may also lead to resistance to sorafenib, for their similar mechanisms [20]. However, the hypothesis does warrant further investigation. Given that anti-tumor activity of metformin as a single agent is limited, investigating the safety and efficacy of metformin acting as adjuvant or neo-adjuvant therapies is an essential task. Further studies should take the complex interactions into account in the design and progress.

Hepatitis B virus is the dominant risk factor for HCC in most areas of Asia, whereas it accounts for only 23% of HCC in the developed Western countries [7], where alcohol-related cirrhosis, hepatitis C virus, and non-alcoholic fatty liver disease are thought to account for the majority of HCC [40]. Our subgroup analyses found a stronger effect of metformin in Asian liver cancer population (HR = 0.37; 95% CI, 0.30-0.47; *p* < 0.001), suggested that metformin might be just sensitive to certain etiological types of liver cancer, which needed to be further confirmed. Stratified analysis by definition of metformin suggested that the beneficial effect on survival lost significance (HR = 0.69; 95% CI, 0.37-1.29; *p* = 0.249) when defined the exposure as taking metformin before cancer diagnosis. In addition to statistical sake, the finding might be explained by the potential metformin-resistance resulted from long-term use before liver cancer diagnosis, as well as the absence of synergistic benefits between metformin and conventional cancer treatments before diagnosis.

DM is not only an important risk factor for HCC occurrence, but also an unfavorable predictor for survival [41]. Subgroup analyses validated the beneficial effect of metformin use when the population was restricted to liver cancer patients with DM (HR = 0.64; 95% CI, 0.42-0.97; *p* = 0.035). Theoretically, adverse effects of DM itself might cover the curative effect of metformin when compared DM patients with non-DM patients. Actually, our subgroup analysis found worse survival (HR = 1.35; 95% CI, 0.99-1.82; *p* = 0.054) in diabetic metformin users when compared with non-diabetic non-users, while the result might also be partially explained by the small patient population (n = 679).

Lots of factors may affect the survival of liver cancer patients, such as age, personal lifestyle, etiology, clinical staging, tumor size, multiple nodules, liver function reserve, initial treatment and so on [42-45]. To avoid these biases, we performed subgroup analyses of adjusted HRs controlling for certain prognostic factors based on the limited information. After adjusting for age, etiology, index of tumor severity and treatment of liver cancer, the beneficial effects on overall survival for metformin use were significant and stable (pooled HR ranged from 0.47 to 0.57). However, in addition to the presence of high heterogeneity and limited patient populations, unmeasurable confounders were also inevitable, resulting in that the observed associations might not necessarily be causal [46]. Thus more studies with sufficient information are needed to clarify these confounders.

Despite our effects to provide a comprehensive and accurate analysis, several limitations in our meta-analysis needed to be addressed, and merited further discussion. First, although we used broad search terms and systematic strategy in multiple databases to identify as many potential studies as possible, only 11 cohort studies fulfilled the inclusion criteria and were included in the final analysis. The limited included studies and small patient population partly contribute to the high heterogeneity across studies. Unfortunately, sensitivity, meta-regression and subgroup analyses all failed to explore the definite sources of heterogeneity. Second, liver cancer patients included in our meta-analysis were in different health states and their prior treatments were also various. Although we found that the beneficial effect for metformin use persisted in DM patients and on analyses after adjusting for tumor severity and treatment, whether the observed benefit could be expanded to a wider range of populations, including non-DM patients and those received certain type of cancer treatment, needed to be determined. Third, most diabetic liver cancer patients in these studies were simultaneously on multiple ADMs, with changes in pharmacotherapy over time. The comparison for metformin users and non-users had doped effects of other ADMs (had their own inherent cancer-modifying effects), led to a biased association between metformin and outcome. However, it was difficult to perform stratified analysis by controlled ADMs or adjust for other ADMs as the related data were lacking. Fourth, adjustments of included studies were inconsistent and incomplete. Although we have performed subgroup analyses of adjusted HRs controlling for several important prognostic factors based on the limited information, such as age, etiology, index of tumor severity and treatment of liver cancer. Some other confounders were failed to control, like cirrhosis, severity and duration of DM, cumulative dose, continuity of drug use, time-related bias, use of concomitant medications (e.g., statins and non-steroidal anti-inflammatory drugs), which would be important to adjust for residual confounding and provide a more indepth understanding of the nature of metformin use [47], while most studies failed to provide these comprehensive information.

In summary, our meta-analysis of observational studies implies that metformin use significantly benefits the survival of diabetic liver cancer patients. The study somewhat strengthens the role of metformin as a potential candidate for chemo-therapy drug in diabetic liver cancer patients. However, limited by the observational study design and above limitations, a causality cannot be drawn. Further prospective studies are needed to confirm the prognostic benefits and to assess the possibility of metformin as an anti-diabetic regimen in the treatment for a wider range of cancer populations.

## MATERIALS AND METHODS

### Literature search

We searched PubMed, Web of Science, Embase, BIOSIS Previews, Cochrane Library and National Institutes of Health database from their inception to 12 May 2016. In order to include more potential literature, our overall search strategy only included terms for metformin (e.g., “metformin” and “biguanide”) and liver cancer (e.g., “liver or hepatic cancer/carcinoma/tumor/neoplasm”, “hepatocellular carcinoma”, “HCC” and “cholangiocarcinoma”). The two terms were connected by logical word “and”, meanwhile the synonyms were connected by “or”. We also screened bibliographies of selected original studies and review articles. There were no language or publication type restrictions. Attempts were made to contact the corresponding authors for additional data.

### Study selection

Citations were merged together in Endnote, version X7 to facilitate management. Study selection was performed by two authors independently, evaluated by title, abstract and full text. Our overall search targeted articles were included if they (i) evaluated a liver cancer patient population, (ii) reported the exposure to metformin or biguanide, and provided effective comparison groups, (iii) evaluated mortality or survival outcome, (iv) reported HRs and corresponding 95% CIs, or provided sufficient data for their estimations. We compared studies on data source, study population, geographic location and information of authors, to try to avoid overlapping patient populations. The most comprehensive or most recent report was given precedence if there were multiple publications from the same population.

### Data extraction

For each of eligible study, information of the first author, publication year, study design, location, data source, time period, study population, definition of metformin exposure, mean follow-up, comparison groups, mean age, gender, total subject, outcomes, HRs and 95% CIs, and adjustment variables were selectively extracted onto piloted structured forms independently by two authors. Any disagreement during study selection or data collection was resolved by consensus, referring back to the original article. Keeping consistent with most of included studies, we used a cut-point for dichotomizing liver cancer patients into users and non-users of metformin in the final analysis about exposure. If several risk estimations were reported in the same article, the most fully adjusted one was chose for overall analysis (e.g., matched cohort was selected over un-matched cohort, multivariate regression was selected over univariate regression), meanwhile, the others might be included in subgroup analyses according to the concrete conditions.

### Quality assessment

The methodological quality of included observational studies was assessed using the NOS [48]. In this scale, studies were judged from three categories: selection (4 stars) and comparability (2 stars) of study groups, and assessment of the outcome of interest (3 stars). Star rating system was used to indicate the quality, with a score from 0 to 9: 0-5 stars as low quality and 6-9 stars as high quality.

### Statistical analysis

Adjusted estimation was given precedence for the quantitative analysis, while crude estimation served as an alternative in case of the adjusted one was unavailable. Missing or incomplete estimations and 95% CIs were tried to calculate using appropriate summary statistics or Kaplan-Meier curves based on published methods [49]. We expressed the summary results as HRs and corresponding 95% CIs in this work to keep consistent with the estimations reported in all the included studies. Heterogeneity among studies was assessed using Cochrane Q test with a significance level of *p* ≤ 0.1, meanwhile quantified by estimated *I²* with a value of > 50% as the standard of significant heterogeneity [50]. When no statistically significant heterogeneity was shown, the Inverse Variance fixed-effects model was used, otherwise a DerSimonian-Laird random-effects model was employed to calculate the pooled estimations [51]. Sensitivity analyses were conducted to assess the robustness of results. Subsequently, meta-regression analyses were performed to evaluate the following potential heterogeneous factors: publication year, location, NOS score and number of total subject. Significant variables (*p* ≤ 0.1) selected by antecedent univariate meta-regression analysis then entered into the multivariable model.

To further explore potential sources of heterogeneity among studies, and validate the result from overall analysis, we performed subgroup analyses by stratifying original studies according to study quality, location, definition of metformin exposure, and different controlled populations. Analyses of adjusted HRs were emphasized on studies controlling for age, etiology (e.g., infected with hepatitis B/C virus, alcoholic and non-alcoholic fatty liver diseases), index of tumor severity (e.g., tumor size, multiple tumors, cancer stage and metastasis), and treatment of liver cancer (e.g., radiofrequency ablation, radiotherapy, sorafenib, transarterial chemoembolization, liver resection), given their modifying effects on metformin activity on prognostic outcomes of liver cancer patients. Publication bias (considered present if *p* ≤ 0.1) was detected for overall analysis using Begg's test and Egger's test [52, 53]. All *p* values were two-sided, and all the statistical analyses were performed using Stata version 12.0 software (StataCorp, College Station, TX, USA).

## References

[1] El-Serag HB, Mason AC, Key C (2001). Trends in survival of patients with hepatocellular carcinoma between 1977 and 1996 in the United States. Hepatology (Baltimore, Md).

[2] Richardson LC, Pollack LA (2005). Therapy insight: Influence of type 2 diabetes on the development, treatment and outcomes of cancer. Nature clinical practice Oncology.

[3] Barone BB, Yeh HC, Snyder CF, Peairs KS, Stein KB, Derr RL, Wolff AC, Brancati FL (2008). Long-term all-cause mortality in cancer patients with preexisting diabetes mellitus: a systematic review and meta-analysis. Jama.

[4] Siegel RL, Miller KD, Jemal A (2015). Cancer statistics, 2015. CA Cancer J Clin.

[5] Giovannucci E, Harlan DM, Archer MC, Bergenstal RM, Gapstur SM, Habel LA, Pollak M, Regensteiner JG, Yee D (2010). Diabetes and cancer: a consensus report. Diabetes care.

[6] Tseng CH (2016). Metformin may reduce oral cancer risk in patients with type 2 diabetes. Oncotarget.

[7] Singh S, Singh PP, Singh AG, Murad MH, Sanchez W (2013). Anti-diabetic medications and the risk of hepatocellular cancer: a systematic review and meta-analysis. The American journal of gastroenterology.

[8] Yin M, Zhou J, Gorak EJ, Quddus F (2013). Metformin is associated with survival benefit in cancer patients with concurrent type 2 diabetes: a systematic review and meta-analysis. The oncologist.

[9] He XK, Su TT, Si JM, Sun LM (2016). Metformin Is Associated With Slightly Reduced Risk of Colorectal Cancer and Moderate Survival Benefits in Diabetes Mellitus: A Meta-Analysis. Medicine.

[10] Stopsack KH, Ziehr DR, Rider JR, Giovannucci EL (2016). Metformin and prostate cancer mortality: a meta-analysis. Cancer causes & control.

[11] Xu H, Chen K, Jia X, Tian Y, Dai Y, Li D, Xie J, Tao M, Mao Y (2015). Metformin Use Is Associated With Better Survival of Breast Cancer Patients With Diabetes: A Meta-Analysis. The oncologist.

[12] Wan G, Yu X, Chen P, Wang X, Pan D, Wang X, Li L, Cai X, Cao F (2016). Metformin therapy associated with survival benefit in lung cancer patients with diabetes. Oncotarget.

[13] Zhang P, Li H, Tan X, Chen L, Wang S (2013). Association of metformin use with cancer incidence and mortality: a meta-analysis. Cancer epidemiology.

[14] Zheng L, Yang W, Wu F, Wang C, Yu L, Tang L, Qiu B, Li Y, Guo L, Wu M, Feng G, Zou D, Wang H (2013). Prognostic significance of AMPK activation and therapeutic effects of metformin in hepatocellular carcinoma. Clinical cancer research.

[15] Nerstedt A, Cansby E, Amrutkar M, Smith U, Mahlapuu M (2013). Pharmacological activation of AMPK suppresses inflammatory response evoked by IL-6 signalling in mouse liver and in human hepatocytes. Molecular and cellular endocrinology.

[16] Sui X, Xu Y, Wang X, Han W, Pan H, Xiao M (2015). Metformin: A Novel but Controversial Drug in Cancer Prevention and Treatment. Molecular Pharmaceutics.

[17] Qu Z, Zhang Y, Liao M, Chen Y, Zhao J, Pan Y (2012). *In vitro* and *in vivo* antitumoral action of metformin on hepatocellular carcinoma. Hepatology research.

[18] Chen TM, Lin CC, Huang PT, Wen CF (2011). Metformin associated with lower mortality in diabetic patients with early stage hepatocellular carcinoma after radiofrequency ablation. Journal of gastroenterology and hepatology.

[19] Bhat M, Chaiteerakij R, Harmsen WS, Schleck CD, Yang JD, Giama NH, Therneau TM, Gores GJ, Roberts LR (2014). Metformin does not improve survival in patients with hepatocellular carcinoma. World journal of gastroenterology.

[20] Casadei Gardini A, Marisi G, Scarpi E, Scartozzi M, Faloppi L, Silvestris N, Masi G, Vivaldi C, Brunetti O, Tamberi S, Foschi FG, Tamburini E, Tenti E (2015). Effects of metformin on clinical outcome in diabetic patients with advanced HCC receiving sorafenib. Expert opinion on pharmacotherapy.

[21] Yang Z, Zhang X, Roberts LR, Chaiteerakij R (2015). Metformin Use Reduces Intrahepatic Cholangiocarcinoma Risk in Patients With Diabetes but Does Not Improve Survival of Cholangiocarcinoma. Gastroenterology.

[22] Chaiteerakij R, Baichoo E, Roberts LR (2013). Metformin use improves survival of cholangiocarcinoma (CC) patients with type II diabetes (DM). Hepatology (Baltimore, Md).

[23] Akmal K, Hassabo H, Botrus G, Shah N, Soliman K, Khalaf R, Li D, Kaseb A, Hassan M (2012). Impact of metformin on HCC prognosis. American Association for Cancer Research.

[24] Currie CJ, Poole CD, Jenkins-Jones S, Gale EA, Johnson JA, Morgan CL (2012). Mortality after incident cancer in people with and without type 2 diabetes: impact of metformin on survival. Diabetes care.

[25] Hassabo HM, Iwasaki M, Soliman K, Abaza Y, Kaseb AO, Torres HA, Li D, Xiao L, Morris JS, Hassan M (2012). Impact of metformin on HCC prognosis in presence and absence of HCV infection. Hepatology (Baltimore, Md).

[26] Graef S, Berhane S, Joey Teng M, Skowronska A, Johnson PJ (2013). The impact of diabetes on HCC. American Society of Clinical Oncology.

[27] Ampuero J, Calle R, Figueruela B, Ferrero P, Suarez E, Romero-Gomez M (2014). Statins and metformin use improve prognosis after diagnosis of hepatocelullar carcinoma. Hepatology (Baltimore, Md).

[28] Jang WI, Kim M-S, Lim JS, Yoo HJ, Seo YS, Han CJ, Park SC, Kay CS, Kim M, Jang HS, Lee DS, Chang AR, Park HJ (2015). Survival Advantage Associated with Metformin Usage in Hepatocellular Carcinoma Patients Receiving Radiotherapy: A Propensity Score Matching Analysis.

[29] Yang Z, Zhang X, Roberts RO, Roberts LR, Chaiteerakij R (2015). Metformin does not improve survival of cholangiocarcinoma in persons with diabetes. Hepatology (Baltimore, Md).

[30] Seo YS, Kim YJ, Kim MS, Suh KS, Kim SB, Han CJ, Kim YJ, Jang WI, Kang SH, Tchoe HJ, Park CM, Jo AJ, Kim HJ (2016). Association of Metformin Use With Cancer-Specific Mortality in Hepatocellular Carcinoma After Curative Resection: A Nationwide Population-Based Study. Medicine.

[31] Sadeghi N, Abbruzzese JL, Yeung SC, Hassan M, Li D (2012). Metformin use is associated with better survival of diabetic patients with pancreatic cancer. Clinical cancer research.

[32] Spillane S, Bennett K, Sharp L, Barron TI (2013). A cohort study of metformin exposure and survival in patients with stage I-III colorectal cancer. Cancer epidemiology, biomarkers & prevention.

[33] Margel D, Urbach DR, Lipscombe LL, Bell CM, Kulkarni G, Austin PC, Fleshner N (2013). Metformin use and all-cause and prostate cancer-specific mortality among men with diabetes. Journal of clinical oncology.

[34] Kumar S, Meuter A, Thapa P, Langstraat C, Giri S, Chien J, Rattan R, Cliby W, Shridhar V (2013). Metformin intake is associated with better survival in ovarian cancer: a case-control study. Cancer.

[35] Skinner HD, Crane CH, Garrett CR, Eng C, Chang GJ, Skibber JM, Rodriguez-Bigas MA, Kelly P, Sandulache VC, Delclos ME, Krishnan S, Das P (2013). Metformin use and improved response to therapy in rectal cancer. Cancer medicine.

[36] Pawlyk AC, Giacomini KM, McKeon C, Shuldiner AR, Florez JC (2014). Metformin pharmacogenomics: current status and future directions. Diabetes.

[37] Pollak M (2012). The insulin and insulin-like growth factor receptor family in neoplasia: an update. Nature reviews Cancer.

[38] Martin-Castillo B, Vazquez-Martin A, Oliveras-Ferraros C, Menendez JA (2010). Metformin and cancer: doses, mechanisms and the dandelion and hormetic phenomena. Cell cycle (Georgetown, Tex).

[39] Groenendijk FH, Mellema WW, van der Burg E, Schut E, Hauptmann M, Horlings HM, Willems SM, van den Heuvel MM, Jonkers J, Smit EF, Bernards R (2015). Sorafenib synergizes with metformin in NSCLC through AMPK pathway activation. International journal of cancer.

[40] Baffy G, Brunt EM, Caldwell SH (2012). Hepatocellular carcinoma in non-alcoholic fatty liver disease: an emerging menace. Journal of hepatology.

[41] Wang YG, Wang P, Wang B, Fu ZJ, Zhao WJ, Yan SL (2014). Diabetes mellitus and poorer prognosis in hepatocellular carcinoma: a systematic review and meta-analysis. PloS one.

[42] Ng KK, Poon RT, Lo CM, Yuen J, Tso WK, Fan ST (2008). Analysis of recurrence pattern and its influence on survival outcome after radiofrequency ablation of hepatocellular carcinoma. Journal of gastrointestinal surgery.

[43] Lencioni R, Cioni D, Crocetti L, Franchini C, Pina CD, Lera J, Bartolozzi C (2005). Early-stage hepatocellular carcinoma in patients with cirrhosis: long-term results of percutaneous image-guided radiofrequency ablation. Radiology.

[44] Takahashi S, Kudo M, Chung H, Inoue T, Ishikawa E, Kitai S, Tatsumi C, Ueda T, Minami Y, Ueshima K, Haji S (2007). Initial treatment response is essential to improve survival in patients with hepatocellular carcinoma who underwent curative radiofrequency ablation therapy. Oncology.

[45] Chen TM, Chang TM, Huang PT, Tsai MH, Lin LF, Liu CC, Ho KS, Siauw CP, Chao PL, Tung JN (2008). Management and patient survival in hepatocellular carcinoma: does the physician's level of experience matter?. Journal of gastroenterology and hepatology.

[46] Johnson JA, Gale EA (2010). Diabetes, insulin use, and cancer risk: are observational studies part of the solution-or part of the problem?. Diabetes.

[47] Walker JJ, Johnson JA, Wild SH (2013). Diabetes treatments and cancer risk: the importance of considering aspects of drug exposure. The lancet Diabetes & endocrinology.

[48] Stang A (2010). Critical evaluation of the Newcastle-Ottawa scale for the assessment of the quality of nonrandomized studies in meta-analyses. European journal of epidemiology.

[49] Guyot P, Ades AE, Ouwens MJ, Welton NJ (2012). Enhanced secondary analysis of survival data: reconstructing the data from published Kaplan-Meier survival curves. BMC medical research methodology.

[50] Higgins JP, Thompson SG, Deeks JJ, Altman DG (2003). Measuring inconsistency in meta-analyses. BMJ (Clinical research ed).

[51] Mantel N, Haenszel W (1959). Statistical aspects of the analysis of data from retrospective studies of disease. Journal of the National Cancer Institute.

[52] Egger M, Davey Smith G, Schneider M, Minder C (1997). Bias in meta-analysis detected by a simple, graphical test. BMJ (Clinical research ed).

[53] Begg CB, Mazumdar M (1994). Operating characteristics of a rank correlation test for publication bias. Biometrics.

